# Mind Cure Matter, Etc.

**Published:** 1888-12

**Authors:** E. D. Babbitt

**Affiliations:** 30 East 14th st., N. Y.


### MIND CURE MATTER, ETC.

Some time ago I made the remark in Hall's Journal of Health, that the most of mankind were able to look only on one side of truth. I am constantly being confirmed in this opinion. Our scientists will at one time declare that light, electricity, magnetism, heat, etc., are real things or fluid essences. At another time they will declare that they are not substances at all, mere nonentities in fact, being only "motions of the ultimate particles of matter."

It doesn't seem to once enter their heads that both of these things are true, as all force in the known realm of things would indicate. The one sidedness of the medical world for centuries in the past, has been the use of course and crude agencies which are so powerful for harm; so weak in their good effects. The spiritual and refined side of nature has been considered of some importance, but on the whole rather mythical and useless. Cutting, slashing, burning, bleeding, binding, blistering and giving fierce, nauseous drugs was the common practice. As a rebound from those extremes came Homeopathy, Hydropathy, Electricity, Eclecticism, Mind Cure, Psychology, Statuvolism and at last the Sun Cure which deals with the mightiest power of the external universe.

If in the past therapeutics has been founded principally in materialism, at the present time an absolute craze for the other extreme of the heavens under the names of Mental Care, Metaphysical Cure, Christian Science, Faith Cure, etc., is seizing upon the people. It is wonderfully important that the refined potencies of the world should be revealed, and that humanity should be vitalized and *mentalized*, so to speak, but it is immensely important also, that we should build upon supreme truth and not ignore the infinite material universe around us. The Mental Science system can show many excellent and some truly intelligent people, and they do a greatly needed thing when they illustrate and exemplify the power of spiritual forces. You may listen to their reasoning for awhile and you will at first say: That is logical, that is reasonable, but stop! One of their leading premises is built upon the most gigantic falsehood in the world! They have taken it for granted, contrary to all known facts that matter is evanescent; that material conditions should be ignored, and that spirit is all in all.

They declare that disease is nothing but imagination or false belief, and that food and external conditions are unimportant, but the truth is, that matter is not evanescent but eternal, so far as all facts of the known universe go to prove. There is no proof in the heavens above or the earth beneath that spirit can act for a moment aside from matter any more than matter can act without spirit, just how the two co-act, I think can be shown, but space is wanting here.

Desiring to get at the constitution of the universe, I have spent many years in searching into the principles of force and atomic action as already explained in my published works, and among other things I have ascertained, in harmony with scientific discovery:

1st. That atoms do positively exist;

2. That their main form and action may be determined;

3. That the basic principles of chemical affinity may be absolutely known.

4. That the exact conditions and processes which produce disease on on the one hand as well as health and harmony on the other, may be determined, and

5. That definite principles of cure may be attained to.

At present, the mass of mankind are too much under the gross material conditions to receive the mental forces actively. *Fineness is power*, when conditions are right, but not only should magnetic and psychic radiations be transmitted to the suffering, but all subtle agencies, such as sunlight, electricity, pure air, food medicines, etc. Fine cures, of course, have often been made through the mental action of others as well as of one's self, but fine cures can often be made in other ways, where mental action is helpless to do good.

Two cases of consumption have been cured by four solar sweat baths each, and a case of lameness of many years standing, was cured in fifteen minutes by concentrating red light upon it. Because I can tell of many marvelous things that have been done by sun baths, must I ignore all other methods? That would be folly and fanaticism.

Mr. J. M. Washburn, in the Social Science Review, committed an error which all mental curists seem to have fallen into! "Sin brought disease ; sin is disease in the soul ; and sin extended into the nerves and the physical body, is the cause of disease in the body."

The word *sin* is not a very scientific one when applied to human conditions, for in a multitude of cases it is disease, not innate depravity that makes the trouble. We can trace what Mr. Washburn calls sin to disease oftener than we can trace disease to sin. Persons who have excitable nerves and an inflamed gastric membrane will frequently have a burning thirst for liquor. Parents and friends will tell them to use their will power mightily and determine to be men. This is mind cure. It sometimes succeeds and persons will become permanently reformed, but alas! how often it fails. The old method which calls it a sin instead of a disease of the bodily organs, does not reach the cause ; the old fires, coming like a flood, sweep the poor sufferer off his feet and he is gone. I have taken several cases, healed the gastric region and toned up the nervous system and they could go into all kinds temptations without desiring stimulus. The same principle is true concerning the excessive animalism that wrecks countless thousands of lives. Pious parents after communicating diseased blood to their children will load them down with meats and other heating foods, perhaps with wines until the hypogastric plexus of nerves is all afire and they are led to commit those acts for which the religionist of the day will doom them to endless destruction. The true philosophy of life teaches just how to tone down these fiery impulses by regulating the physical conditions and using also psychological and mental forces where it is practicable. To build only on mental forces is to build on falsehood and in many cases to do great injury. There is no true upbuilding of mental and spiritual conditions without the nicest attention to bodily conditions. The Christian Scientists inveigh against the dangers of wrong thinking and I trust they will try to correct their own wrong thinking.

Perhaps it will be well to end my serious talk on this subject with a humorous article by J. B. S. King in the Medical Visitor of Chicago. He says "A Christian Scientist, whose time was fully occupied in thinking about the unreality of disease at $2 per think, once treated a highly unappreciative man for a chronic nervous affection of a very painful character." After this man had depleted his purse by spending $40 thus, without any improvement he desired to know when he should begin to get better.

"Then the Christian Scientist waxed wroth and said : O you of little faith ! Know that you would already have been cured, if you had believed me, when I told you that your pain was not real. Pain and suffering do not exist ; they are merely phantasms of the brain. There is no such thing as matter," continued he with such emphasis that it rattled some silver dollars in his pocket, "none, whatever : the only real thing is thought. All this is too subtle for your commonplace mind, and hence I can do nothing more for you ; you had better go and fill your coarse, unappreciative system with drugs."

Then a vision of $40 that had vanished, and of pain that vanished not, came before the mind of that long suffering man, and he arose, and he took that Christian Scientist, and he mopped the floor with him, smiting him sore upon the head and back, so that when he was through, congestions, abrasions, contusions, incipient ecchymoses and epistaxis were among the phenomena presented by his Christian countenance.

"There is no real suffering, said the Unappreciating man, with withering scorn. The bruises on your alleged head are entirely hypothetical; the choking I gave you was simply an idea of mine, and a devilish good idea too ; the pain which you feel is merely an intellectual fantasy, and your nose bleed is only one of the ideal conceptions of the cerebral mass. Believe these things not to exist and they vanish. Good day, sir." And the patient departed.

"Damn that man, growled the Christian, as he limped to a sink."

30 East 14th st., N. Y. E. D. Babbitt, M. D.

